# Supplementary material for: BET Inhibitor JQ1 Attenuates Feline Leukemia Virus DNA, Provirus, and Antigen Production in Domestic Cat Cell Lines
Source: Viruses. 2023 Aug 31;15(9):1853. doi: 10.3390/v15091853 (PMC10535802; doi:10.3390/v15091853)
Supplement: Supplementary file 1 [file viruses-15-01853-s001.zip › viruses-2570184-supplementary.pdf]

Table S1. Percent identity matrices for BET proteins BRD2, BRD3, and BRD4 in selected mammalian species. Created using Clustal Omega 2.1 [57].

Sequence No.	Protein - Species	% Sequence Similarity with Protein of Sequence No.		
		1	2	3
1	BRD2 - *Felis catus*			
2	BRD2 - *Mus musculus*	96.87		
3	BRD2 - *Homo sapiens*	98.63	96.61	
**Sequence No.**	**Protein - Species**	**% Sequence Similarity with Protein of Sequence No.**		
		**4**	**5**	**6**
4	BRD3 - *Felis catus*			
5	BRD3 - *Mus musculus*	93.11		
6	BRD3 - *Homo sapiens*	93.79	95.44	
**Sequence No.**	**Protein - Species**	**% Sequence Similarity with Protein of Sequence No.**		
		**7**	**8**	**9**
7	BRD4 - *Felis catus*			
8	BRD4 - *Mus musculus*	95.22		
9	BRD4 - *Homo sapiens*	97.13	95.74	

Table S2. Percent identity matrices for ET domains of BET proteins BRD2, BRD3, and BRD4 in selected mammalian species. Created using Clustal Omega 2.1 [57].

Sequence No.	Protein Domain - Species	% Sequence Similarity with Protein Domain of Sequence No.		
		1	2	3
1	BRD2 - *Felis catus*			
2	BRD2 - *Mus musculus*	100		
3	BRD2 - *Homo sapiens*	100	100	
**Sequence No.**	**Protein Domain - Species**	**% Sequence Similarity with Protein Domain of Sequence No.**		
		**4**	**5**	**6**
4	BRD3 - *Felis catus*			
5	BRD3 - *Mus musculus*	100		
6	BRD3 - *Homo sapiens*	100	100	
**Sequence No.**	**Protein Domain - Species**	**% Sequence Similarity with Protein Domain of Sequence No.**		
		**7**	**8**	**9**
7	BRD4 - *Felis catus*			
8	BRD4 - *Mus musculus*	100		
9	BRD4 - *Homo sapiens*	100	100	

Figure S1. Effects of (+)-JQ1 over time on percent viability of 3201 cell cultures challenged with FeLV.

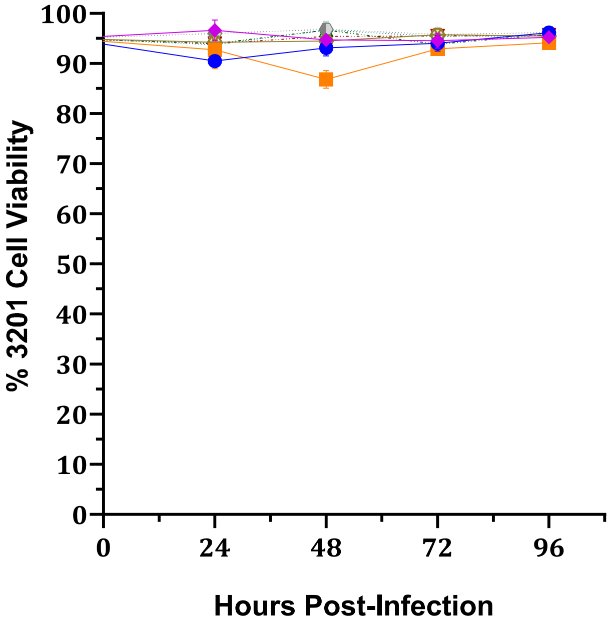

No significant changes in % 3201 cell viability were observed at any at any measured (+)-JQ1 concentration or timepoint compared to controls. D(-)V(-)T(-) indicates FeLV-unexposed, untreated cells. D(+)V(+)T(-) indicates FeLV-exposed, DMSO vehicle-treated cells. D(+)V(-)T(-) indicates FeLV-unexposed, DMSO vehicle-treated cells. One-way ANOVA with Holm-Šídák multiple comparisons test. Error bars represent ± SEM; $n = 3$ biological replicates. * $p < 0.05$; ** $p < 0.01$; *** $p < 0.001$; **** $p < 0.0001$
